# Molecular Structure Effect of a Self-Assembled Monolayer on Thermal Resistance across an Interface

**DOI:** 10.3390/polym13213732

**Published:** 2021-10-28

**Authors:** Lijian Song, Youchen Zhang, Weimin Yang, Jing Tan, Lisheng Cheng

**Affiliations:** 1 College of Mechanical and Electrical Engineering, Beijing University of Chemical Technology, Beijing 100029, China; song_li_jian@163.com (L.S.); yangwm@mail.buct.edu.cn (W.Y.); tanj@mail.buct.edu.cn (J.T.); 2 State Key Laboratory of Organic-Inorganic Composites, Beijing University of Chemical Technology, Beijing 100029, China

**Keywords:** molecular dynamic simulation, coarse-grained, thermal resistance, self-assembled monolayer, PEG

## Abstract

Understanding heat transfer across an interface is essential to a variety of applications, including thermal energy storage systems. Recent studies have shown that introducing a self-assembled monolayer (SAM) can decrease thermal resistance between solid and fluid. However, the effects of the molecular structure of SAM on interfacial thermal resistance (ITR) are still unclear. Using the gold–SAM/PEG system as a model, we performed nonequilibrium molecular dynamics simulations to calculate the ITR between the PEG and gold. We found that increasing the SAM angle value from 100° to 150° could decrease ITR from 140.85 × 10^−9^ to 113.79 × 10^−9^ m^2^ K/W owing to penetration of PEG into SAM chains, which promoted thermal transport across the interface. Moreover, a strong dependence of ITR on bond strength was also observed. When the SAM bond strength increased from 2 to 640 $\mathrm{kcal}\cdot {\mathrm{mol}}^{-1}{Å}^{-2}$, ITR first decreased from 106.88 × 10^−9^ to 102.69 × 10^−9^ m^2^ K/W and then increased to 123.02 × 10^−9^ m^2^ K/W until reaching a steady state. The minimum ITR was obtained when the bond strength of SAM was close to that of PEG melt. The matching vibrational spectra facilitated the thermal transport between SAM chains and PEG. This work provides helpful information regarding the optimized design of SAM to enhance interfacial thermal transport.

## 1. Introduction

Thermal energy storage is one of the most effective and efficient approaches to realize optimized energy utilization since it bridges the gap between energy supply and energy demand [1,2]. This is especially important for the use of weather-dependent solar energy [3]. For a thermal energy storage system, thermal conductivity is one of the most critical factors that determine the effectiveness and performance of the system [1]. A higher thermal conductivity can speed up the process of thermal energy storage and release. Thermal conductivity is inversely correlated to thermal resistance. The total thermal resistance of the thermal energy storage system generally consists of contributions from the storage medium itself and various interfacial thermal resistances (ITRs). Among these contributions, the interfacial thermal resistance (ITR) between dissimilar materials, such as the fluidic medium and the solid, plays a dominant role.

In the past few years, efforts have been made to find out strategies to reduce ITR between the solid and fluid. Among these strategies, one of the most promising approaches is to anchor a self-assembled monolayer to the solid surface [4]. With this approach, researchers have focused on modifying the interface with the alkanethiol SAMs, X–(CH_2_)n–SH, where X represents –COOH, –OH, –CH_3_, –NH_2_, and so on. Most recently, several types of SAMs have been proposed to improve thermal conductance. These SAMs include: cetyltrimethylammonium bromide (CTAB) and polyethylene glycol (PEG) grafted on a Au surface [5,6], poly (acrylic acid) (PAA), polyacrylamide (PAM), polyvinyl alcohol (PVA),poly (acrylic acid)/polyethyleneimine (PAA/PEI) SAMs layer-by-layer-anchored on lithium cobalt oxide [7], and phosphonate-functionalized azastibazolium π-electron (PAE) SAMs on Pt electrodes [8].

These studies have investigated the effects of various characteristics of SAMs on the interfacial thermal conductance from two different points of view: **(1) considering accurate information about SAM**, including SAM coverage [4,9,10], length of SAMs [4,11,12,13], distinct functional group [9,13,14,15,16,17], heterogeneous fin structure [18], vibrational spectral overlapping [9], nanoscale roughness [19,20,21], and so on. Huang et al. [16] investigated interfacial thermal conductance (ITC) across the interface of water and gold tailored with –CH_3_SAM, –OHSAM, and –COOHSAM. The atomic interactions were decomposed into Lennard–Jones (LJ) and electrostatic interactions. They found a collaborative effect from the electrostatic and LJ interactions, and both electrostatic and LJ interactions decreased the ITR between the gold and water. Ju et al. [22] studied the thermal transport properties between water and gold coated with a SAM. They found that the ITR between gold and water remained constant regardless of the density of SAM. Lu et al. [10] investigated the effects of packing density and alkyl-chain length of SAMs on the ITC between the gold and polyethylene (PE) interface by experiments and molecular dynamics (MD) simulations. They concluded that, for a SAM with a high packing density, when the chain of the SAM reached a certain length, the ITC became stable. **(2) Changing various structural parameters of SAM, such as mass and bond strength**. Hung et al. [16] changed the bond strength to investigate ITC between water and SAM. They found that interfacial thermal conductance between SAM and water $G_{SAM/water}$ strongly depended on the bond strength of SAM. Sun et al. [23] performed nonequilibrium molecular dynamics (NEMD) simulations to investigate the thermal conductance of the gold–SAM interface, and they found that the strength of the gold–SAM bond can affect the thermal conductance. Their results indicated that the thermal flow can be facilitated by strengthening the gold–SAM bonds because stronger bonds lead to smaller anharmonicity at the gold–SAM interface, hence decreasing thermal energy dissipation. Zhang et al. [24] found that increasing the strength of the force constant of dihedral interactions reduces the rotation of the polymer segments and increases the radius of gyration ($R_{g}$). According to their results, a larger $R_{g}$ contributes to a larger thermal conductivity. Jiahao Chen et al. [25] noted that SAM tilt angle, defined as angle between flat substrates headgroup atoms, dominated intermolecular forces of SAM chains and influenced its interfacial transport performance. Damien Thompson et al. [26] suggested that the tilt angle of the headgroup should be optimized to reduce steric repulsions and maximize SAM packing. These findings suggest that the molecular structure parameters influence material thermal properties and interfacial transport behaviors.

In these various types of SAM, PEG SAM was the most popular and easily synthesized in an aqueous solution. Its detailed structure and dynamic have been investigated. Jiaqi Lin et al. [27] studied the effect of grafting density, chain length, and particle surface curvature on the properties of PEG on gold surfaces. They found long chain length could substantially decrease the solubility of hydrophobic materials. Fengxuan Jiao et al. [28] implemented coarse-grained molecular dynamics simulations to study interactions of gold with PEG SAM. It was found that concentrations of gold impacted the SAM surface tension. Sutapa Dutta [29] attached bPEG into PEG via a covalent bond, and they concluded that the repulsion between bPEG and PEG had an effect on availability of biotins molecule. Albert J. Power et al. [30] suggested that torsional angles of SAM affected SAM chain conformations and dynamics behaviors.

However, the exact dependence of ITR on the structure parameter of PEG SAM is still insufficient. Therefore, we aim to achieve insight into the influence of the molecular structure of SAM on ITR by NEMD simulations in this work. We took SAM grafted on the gold surface as an example to explore the regularity between SAM structural parameters and ITR. We considered the bond and angle potential parameters, including the equilibrium angle and the bond force constant. In addition, we calculated the density profile, interfacial adhesion energy, radial distribution function (RDF), vibrational density of state (VDOS), and overlapping factor between SAM and the PEG. These results provided a structure–activity relationship for interfacial thermal transport and showed the importance of molecular engineering in heat transfer.

## 2. Material and Molecular Modelling

**Figure 1:**
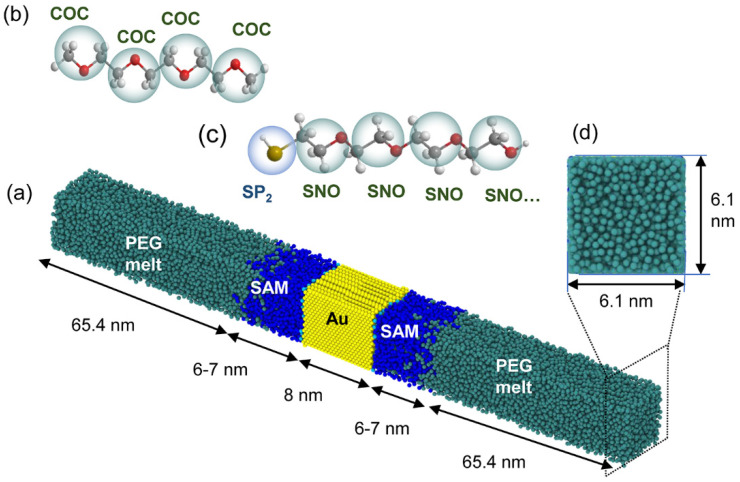
Schematic of the simulation system: (**a**) the system comprises three parts: PEG melt layer (cyan), self-assembled monolayer (SAM) layer (blue), and inorganic gold layer (yellow). (**b**) The coarse-grained (CG) model of medium PEG. (**c**) The CG model of PEG in the brush has 32 beads and it is grafted on the gold layer with the SP_2_ bead. (**d**) The cross-sectional dimensions of the system are 6.1 nm × 6.1 nm.

Figure 1 shows the schematic of the system comprising three layers. The organic layer was made of PEG at each end of the system. The SAMs were grafted on the inorganic gold layer, which was in the middle of the system. The cross-section of the system had a size of 6.1 nm × 6.1 nm (Figure 1d). The thickness of the gold layer was 8 nm, which was enough to avoid the size effect [31]. The thickness of the SAM ranged from 6 to 7 nm due to different equilibrium angles of the SAM chains. To eliminate the size effect of PEG, we calculated thermal resistance for different PEG sizes that were much larger than the mean free path of PEG. The ITR between the gold and PEG melt was calculated in the directions parallel to the heat flow. The results showed that the size effect was evident in the horizontal direction but not apparent in the vertical direction (Figure S1 in the Supplementary Materials), as depicted in Figure 1a.

The coarse-grained (CG) model was applied throughout the simulation, considering the computational cost. The inorganic material layer contained 8000 gold beads, and the Morse pairwise potential for the pairwise interaction between the gold CG beads was employed from previous works [32]. The properties of gold, such as density, kinetic energy, and total potential energy at 300 K, were reproduced from the CG model. The results showed that the density of gold in the CG model is 19.93 g/cm^3^ at 300 K, similar to that of the pure gold crystal. Additionally, we compared the VDOS of the all-atom gold with that of our CG model, and the result also showed no significant difference between the all-atom and CG models (Figure S2), which suggests our CG model was reasonable for heat transfer [33].

As shown in Figure 1b, each PEG chain is comprised of four beads named COC. The SAM chains consist of SP_2_ and SNO beads. The SP_2_ bead represents “Au-S”, and SNO bead represents a “–CH_2_–CH_2_–O–” or “CH_2_–CH_2_–OH” group. We chose 32 beads in a SAM chain to avoid the chain length effect on the thermal resistance [10]. During the simulations, SAMs were grafted to the left- or right-side surface of the gold layer through covalent bonds with a grafted density of 3.4 chains/nm^2^. The simulation parameters of the PEG and SAM used in this work were obtained from the literature [34].

The topology and initial configuration were constructed by the Moltemplate and Packmol packages, respectively. All MD simulations were performed using the Large-scale Atomic/Molecular Massively Parallel Simulator (LAMMPS) package in a periodic boundary condition along three dimensions with a time step of 2 fs. The trajectory data were visualized using OVITO 3.0 [35]. First, we performed an energy minimization to overcome the bad configuration containing highly overlapped atoms. Then, the entire system was equilibrated at 420 K under atmospheric pressure using a constant pressure and constant temperature (NPT) ensemble for 4 ns. After NPT equilibration, a canonical (NVT) ensemble was performed for 4 ns to reach a steady state. Then, we performed an NVE ensemble for 1 ns to check the conservation of the system’s total energy and ensure that the temperature no longer drifted.

**Figure 2:**
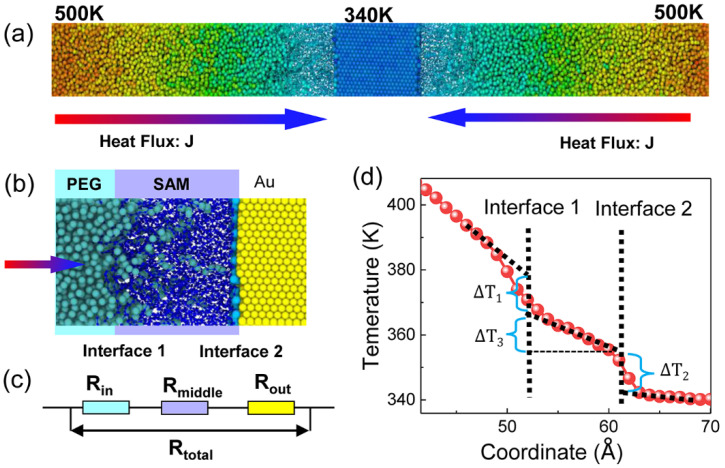
(**a**) Heat flux J applied at the ends of the system, generating temperature distribution along with the system. The heat source and heat sink temperatures were controlled at 500 K and 340 K, respectively. (**b**) Locations of interface “1” and interface “2.” (**c**) Serial model of the total thermal resistance between gold and PEG. (**d**) Temperature profile near the interface and calculation of temperature drop.

Subsequently, an NEMD simulation was executed. In Figure 2a, the heat source and heat sink were applied to the PEG and gold layers, respectively, with a thermal bath thickness of 2.34 nm. Langevin thermostats released energy to the heat source and heat sink with a temperature of 500 K and 340 K for 4 ns, respectively. The temperature was controlled with a relaxation time of 0.2 ps. The effect of the relaxation time on thermal properties was tested from 0.01 to 1 ps, and 0.2 ps was chosen (Figure S3). By holding the two regions at different temperatures, heat flux was formed from the PEG to the gold along the x direction. During the simulations, we divided the simulation box into several slabs along the heat flow direction, and each slab had a width of 4.67 Å and 230–240 atoms to ensure precise temperature statistics.

After the steady state was reached, heat flux, density distribution, and temperature profiles were collected. The production process was repeated three times, and the thermal resistance was calculated and averaged to reduce the deviation. We calculated the ITR using the serial model. The R_total_ between PEG and gold was defined as follows:

(1) $$R_{\mathrm{total}}=R_{\mathrm{in}}+R_{\mathrm{middle}}+R_{\mathrm{out}}$$

(2) $$R_{\mathrm{in}}=\frac{\Delta T_{1}}{J}$$

(3) $$R_{\mathrm{out}}=\frac{\Delta T_{2}}{J}$$

(4) $$R_{\mathrm{middle}}=\frac{\Delta T_{3}}{J}$$

where $R_{\mathrm{in}}$ was the ITR at interface 1 described in Figure 2b,c. The thermal resistance prevented heat flow from PEG to the SAM. Similarly, $R_{\mathrm{out}}$ represented the heat flow from the SAM to gold at interface 2. For simplification, the thermal resistance of SAM and partial PEG molecules was collectively named as $R_{\mathrm{out}}$. In Figure 2d, temperature drop, $∆T_{i}$ (*i* = 1, 2, 3), was extracted at interface 1 and interface 2:

(5) $$x_{PEG/SAM}=\frac{1}{2}\times \left(\frac{\int x\rho_{PEG}dx}{\int \rho_{PEG}dx}+\frac{\int x\rho_{SAM}dx}{\int \rho_{SAM}dx}\right)$$

(6) $$x_{\mathrm{gold}/SAM}=\frac{1}{2}\times \left(\frac{\int x\rho_{gold}dx}{\int \rho_{\mathrm{gold}}\mathrm{dx}}+\frac{\int x\rho_{\mathrm{SAM}}\mathrm{dx}}{\int \rho_{\mathrm{SAM}}\mathrm{dx}}\right)$$

where $X_{\mathrm{PEG}/\mathrm{SAM}}$ was the position of interface 1 and $X_{\mathrm{gold}/\mathrm{SAM}}$ was the position of interface 2. $\rho_{\mathrm{PEG}}$, $\rho_{\mathrm{SAM}}$, and $\rho_{\mathrm{gold}}$ were the densities of PEG, SAM, and gold, respectively. It should be noted that our results intend to provide a qualitative trend rather than quantitative data.

## 3. Results and Discussion

From the force field energy function according to Equation (7), $E_{\mathrm{angle}}$ and $E_{\mathrm{bond}}$ tend to occupy a significant proportion of all function items and are demonstrated to be substantial in polymer thermal transport [36]. Hence, we focused on two main structural factors: SAM equilibrium angle of backbone $\theta_{\mathrm{eq}}$ and bond stretching $K_{b}$ defined in Formulas (8) and (9). The $\theta_{\mathrm{eq}}$ angle was among three SNO beads. Similarly, the $K_{b}$ bond strength was between two SNO beads.

We applied the concept of $\theta_{\mathrm{eq}}$ and $K_{b}$ to characterize the ductility and stiffness of SAM. We first studied the angle effect by fixing the value of $K_{b}$ at 20 $\mathrm{kcal}\cdot {\mathrm{mol}}^{-1}{Å}^{-2}$ and the $\theta_{\mathrm{eq}}$ varied from 100° to 150°. Likewise, bond effect was investigated with $K_{b}$ varied from 2 to 640 $\mathrm{kcal}\cdot {\mathrm{mol}}^{-1}{Å}^{-2}$ and $\theta_{\mathrm{eq}}$ restricted at 130°.

(7) $$E_{\mathrm{total}}=E_{\mathrm{bonded}}+E_{nonbonded}=(E_{angle}+E_{bond}+E_{dihed})+(E_{vdw}+E_{coul})$$

(8) $$E_{angle}=\sum_{angles}K_{a}{\left(\theta -\theta_{eq}\right)}^{2}$$

(9) $$E_{bond}=\sum_{bonds}K_{b}{\left(r-r_{eq}\right)}^{2}$$

(10) $$E_{dihed}=\sum_{i=1,m}K_{i}[1+\cos(n_{i}\phi -d_{i})]$$

where $E_{\mathrm{total}}$ is the total energy of the system; it comprises angle bending ($E_{\mathrm{angle}}$), bond stretching ($E_{\mathrm{bond}}$), and dihedral torsion terms ($E_{\mathrm{dihedral}}$) for bonded interactions, Lennard–Jones ($E_{\mathrm{vdw}}$) and Coulomb ($E_{\mathrm{coul}}$) terms for nonbonding interactions. This study did not consider Coulomb interactions because the heat carriers were mainly phonons rather than electrons across the nonmetal interface.

### 3.1. Angle Effect of SAM on $R_{\mathrm{total}}$

**Figure 3:**
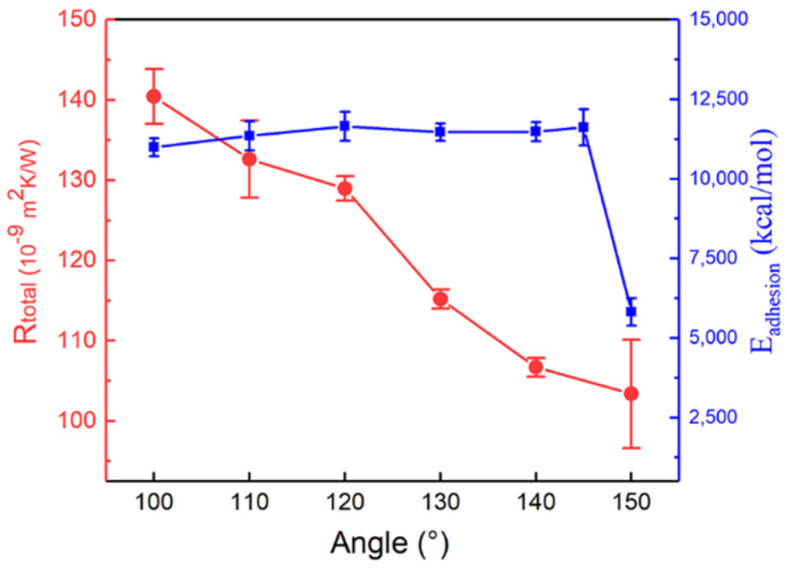
The $R_{\mathrm{total}}$-dependent trend of brush angle (red line). The variation tendency of $E_{adhesion}$ with different SAM angles (blue line).

Figure 3 shows a marked decreasing trend in $R_{\mathrm{total}}$ with an increasing SAM angle. Previous research suggested that, the stronger the interfacial adhesion energy between self-assembled monolayers and soft materials, the less ITR [18]. To further verify this conclusion, we calculated interfacial adhesion energy using the following formula [7]

(11) $$E_{adhesion}=\left|E_{total}-E_{\mathrm{gold}-SAM}-E_{PEG}\right|$$

where $E_{adhesion}$ is adhesion energy between PEG melt and SAM, $E_{total}$ is the total potential energy of the whole system at equilibrium state, $E_{gold-SAM}$ is the energy of gold, and SAM without the PEG. $E_{PEG}$ is the energy of the PEG without the gold–SAM. These energy terms were calculated based on equilibrium molecular dynamic simulation at an average temperature of 420 K with a time step 1 fs.

The ITR decreased with SAM angle, while the $E_{adhesion}$ did not change significantly. The $E_{adhesion}$ initially remained a steady state from 100° to 145°, finally dramatically reduced at 150°. This result indicated that interfacial energy would not exhibit a perfect positive correlation with interfacial thermal resistance.

**Figure 4:**
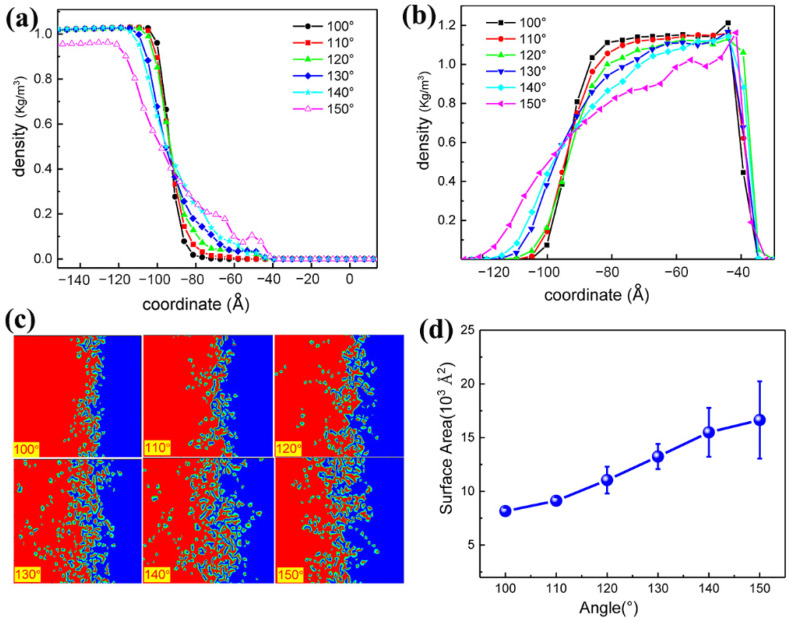
Microstructure of the interface: (**a**,**b**) density profiles of each component perpendicular to the interface for the PEG and SAM, respectively. (**c**) Surface morphology of the contact area between the PEG (blue color) and SAM (red color) for various equilibrium angles. (**d**) An interfacial area as a function of the equilibrium angle.

To clarify the cause of $R_{\mathrm{total}}$ changes triggered by SAM angle, we investigated surface morphologies between the PEG and SAM. From Figure 4a,b, the density of PEG molecules from −100 Å to −40 Å and that of SAM molecules from −120 Å to −90 Å increased. This result led us to speculate that when the SAM angle increased, part of PEG molecules penetrated the space of SAM molecules, and then diminished the distance between SAM and PEG molecules.

**Figure 5:**
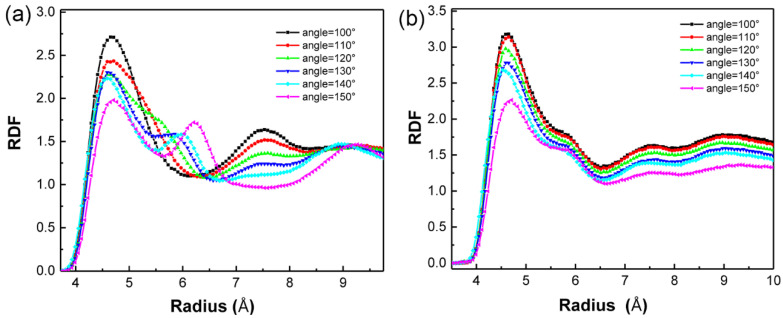
Radial distribution function (RDF) of (**a**) PEG and (**b**) SAM.

In Figure 4c, qualitative studies showed that the contact region enlarged as the angle increased. However, the contact area had no detectable differences when the angle value exceeded 130°. We quantitatively estimated the surface contact area by computing the surface area between PEG melt and SAM using the “construct surface mesh” command in OVITO [37]. This command has its algorithm for controlling a probe sphere to capture the surface details of PEG. In all studies, the radius of the probe sphere was adopted as 4.3 Å according to the first peak position of the RDF curve (Figure 5b). Figure 4d showed that the interfacial contact area was positively correlated with the angle. It can be concluded that, when the SAM angle increases, PEG penetrates SAM, increasing the contact area, which promotes the heat propagation between the PEG melt and SAM molecules. Thus, the $R_{\mathrm{total}}$ between gold and PEG melt decreases.

We investigated the RDF to interpret results and gained more information about the surface structure between PEG and SAM.

(12) $$g(r)=\frac{n(r)}{4\pi r^{2}\rho_{0}\delta_{r}}$$

where n(r) is the number of atoms in a shell with a thickness $\delta_{r}$ at distance r from the reference atom, and $\rho_{0}$ is the average atom number density.

The atomic distribution at the interface played a dominant role in interfacial thermal transport. Hence, the shell and reference atoms were confined around the interface with 5–6 nm. From Figure 5a,b, the RDF of SAM beads showed that the first peak gradually decreased. The RDF of PEG beads also showed the same trend. That is because PEG penetrates SAM molecules and separates atoms. It should be noted that, in Figure 5a, the second peak gradually increased during the increment of the angle, which indicated that the beads in the PEG chains became long-range ordered as the angle value increased. The regular structure may promote heat transportation.

### 3.2. Bond Strength Effect of SAM on $R_{\mathrm{total}}$

**Figure 6:**
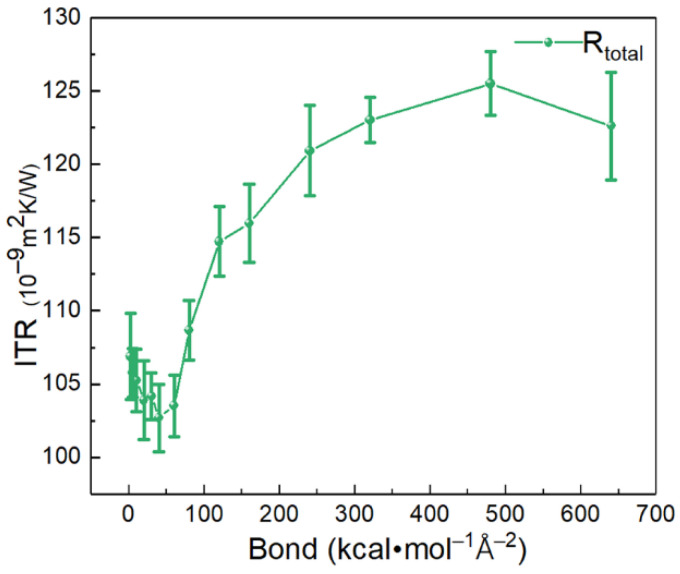
Interfacial thermal resistance (ITR) under various bond strengths of SAM.

As the SAM acts as a channel between the hard and soft materials, revealing the relationship between the ITR and SAM bond strength is essential. We varied the bond strength $K_{b}$ from 2 to 640 $\mathrm{kcal}\cdot {\mathrm{mol}}^{-1}{Å}^{-2}$ by fixing the equilibrium angle $\theta_{\mathrm{eq}}$ at 130°. Notably, this range covered the bond strength of PEG (20.315 $\mathrm{kcal}\cdot {\mathrm{mol}}^{-1}{Å}^{-2}$). Figure 6 showed that $R_{\mathrm{total}}$ decreased with the bond strength less than 30 $\mathrm{kcal}\cdot {\mathrm{mol}}^{-1}{Å}^{-2}$ and then increased rapidly until $K_{b}$ reached 240 $\mathrm{kcal}\cdot {\mathrm{mol}}^{-1}{Å}^{-2}$. Finally, the curve became stable.

To establish the mechanism for the effect of bond strength on ITR, we calculated the VDOS of PEG and SAM beads, respectively. The VDOS was computed by performing the Fourier transform of the velocity autocorrelation function described as

(13) $$VDOS(\omega )=\frac{1}{k_{B}T}\sum_{i}m_{i}\int_{-\infty }^{+\infty }\int_{-\infty }^{+\infty }\vec{v_{i}}(t)\cdot \vec{v_{i}}(t+\tau )e^{i\omega t}dt\;d\tau$$

where $v_{i}$ is the atomic velocity of atom I, T is the average temperature during the dynamic equilibrium process, and $k_{B}$ is the Boltzmann constant.

**Figure 7:**
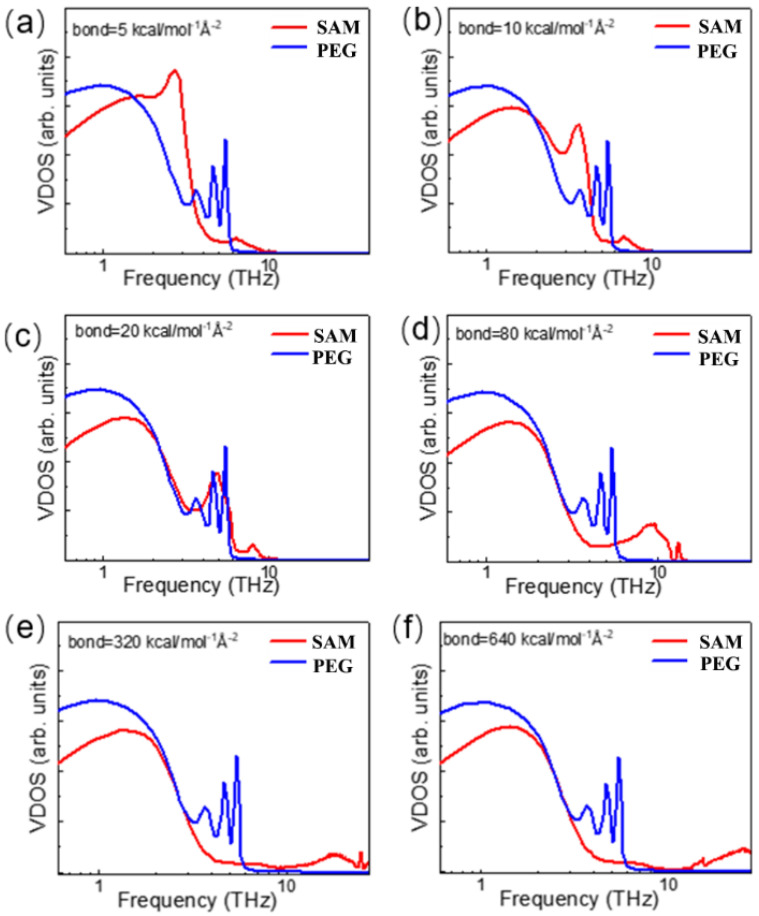
Vibrational density of state (VDOS) profiles of PEG and SAM beads within the interface region at bond strengths of (**a**) 5 $\mathrm{kcal}\cdot {\mathrm{mol}}^{-1}{Å}^{-2}$, (**b**) 10 $\mathrm{kcal}\cdot {\mathrm{mol}}^{-1}{Å}^{-2}$, (**c**) 20 $\mathrm{kcal}\cdot {\mathrm{mol}}^{-1}{Å}^{-2}$, (**d**) 80 $\mathrm{kcal}\cdot {\mathrm{mol}}^{-1}{Å}^{-2}$, (**e**) 320 $\mathrm{kcal}\cdot {\mathrm{mol}}^{-1}{Å}^{-2}$, and (**f**) 640 $\mathrm{kcal}\cdot {\mathrm{mol}}^{-1}{Å}^{-2}$.

Figure 7 shows that the VDOS of the PEG is independent of the bond strength. In other words, changing SAM bond strength $K_{b}$ does not change the vibration frequency distribution of PEG melt. Meanwhile, the VDOS of SAM changed significantly with their bond strength and showed a right shift trend. This is reasonable because the vibration frequency is roughly proportional to $\sqrt{K_{b}/m}$ [23]. As the bond strength increased, the first peak of VDOS in the SAM no longer changed until the value of $K_{b}$ was up to 30 $\mathrm{kcal}\cdot {\mathrm{mol}}^{-1}{Å}^{-2}$. The second peak appeared sequentially and was shifted to the high frequency’s direction.

To quantify the VDOS match between PEG and SAM, the overlap factor S [38] was defined as follows:

(14) $$S=\int_{0}^{\infty }\min\;\{{VDOS}_{PEG}(\omega ),{VDOS}_{SAM}(\omega )\}\;d\omega$$

where ${VDOS}_{PEG}(\omega )$ and ${VDOS}_{SAM}(\omega )$ are the VDOS of PEG and SAM, respectively.

**Figure 8:**
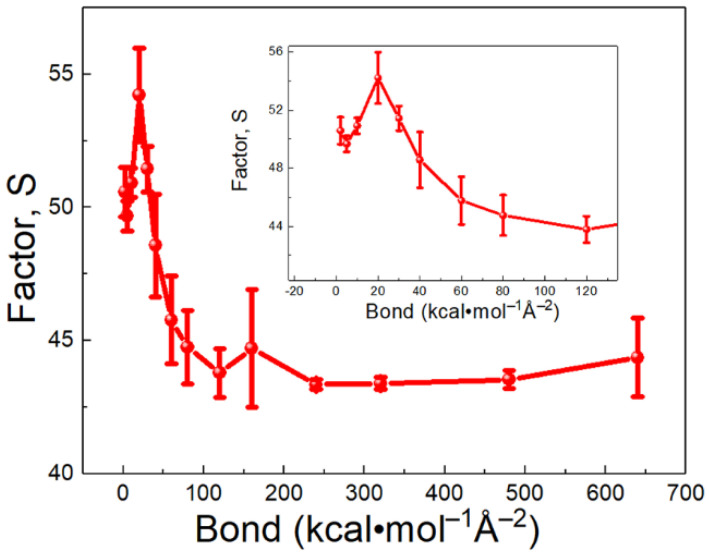
Overlap factor S for beads in PEG medium and brush as a function of bond strength.

According to Figure 8, the trend of overlap factor can explain the observed dependence of $R_{\mathrm{total}}$ on the bond strength. The overlap factor had three stages: (1) when the bond strength increased from 2 to 30 $\mathrm{kcal}\cdot {\mathrm{mol}}^{-1}{Å}^{-2}$ and the factor S increased. In this stage, the bond strength of SAM was close to that of PEG. Molecules between SAM and PEG were easily resonated. Hence, the ITR showed a decreasing trend (Figure 8). (2) When the bond strength increased from 30 to 240 $\mathrm{kcal}\cdot {\mathrm{mol}}^{-1}{Å}^{-2}$ and the factor S showed an opposite trend with the first stage. In this stage, vibration mismatch between PEG and SAM contributed to the increasing trend of the $R_{\mathrm{total}}$. (3) When the bond strength increased from 240 to 640 $\mathrm{kcal}\cdot {\mathrm{mol}}^{-1}{Å}^{-2}$ and the factor S no longer changed. Thus, ITR became stable. These results demonstrate that the ITR can be controlled by adjusting the stiffness of SAMs. As organic materials and SAMs are structurally similar, the ITR between SAMs and organic materials might reach a minimum value.

## 4. Conclusions

We studied the effect of the bond strength and equilibrium angle of SAM on ITR by CG NEMD simulations. We demonstrated that ITR decreased from 140.85 to 113.79 (10^−9^ m^2^ K/W) with the equilibrium angle ranging from 100° to 150°. To better understand the mechanism for the effect of the equilibrium angle on ITR, we addressed the study from the perspective of energy and molecular structure. On the one hand, we calculated the adhesion energy between the PEG and SAM and found that the adhesion energy at the interface could not explain the increasing trend of ITR. On the other hand, the density distribution and RDF indicated that the molecules of the PEG and SAM at their interface became disordered. This meant that more PEG molecules penetrated SAM and promoted thermal transport. We also found that the bond strength of SAM had a significant influence on ITR due to the matching degree between their vibrational spectra. These results provide insights into the mechanisms of interfacial heat transport. This work would be helpful to design and select novel self-assembled monolayers to reduce thermal resistance at the interface between inorganic solids and the organic fluids.

## Data Availability

The data presented in this study are available on request from the corresponding author.

## Supplementary Materials

The following are available online at https://www.mdpi.com/article/10.3390/polym13213732/s1. Figure S1: (a) Schematic representation under different lengths of the system. L is the half-length of the system. gold (yellow), PEG medium (cyan), PEG brush (blue). (b) Length effect on thermal resistance between PEG medium and PEG brush. After L reaches 326 Å, the ITR between PEG medium and PEG brush is stable. (c) Schematic representation of the system with different widths along the z direction. (d) Width effect on the thermal resistance between PEG medium and PEG brush. ITR showed few size effect along the width direction, Figure S2: Normalized vibrational power spectral of Au substrate of all-atom model and our coarse-grained model, Figure S3: Effect of different relaxation times in Langevin thermal bath on ITR between PEG medium and PEG brush, Table S1: Nonbonding interactions parameters of the system in the CG model, Table S2: Bonding parameters of the system in the CG model, Table S3: Angle parameters of the system in the CG model, Table S4: Dihedral parameters of the system in the CG model.
