# Supplementary material for: Molecular Structure Effect of a Self-Assembled Monolayer on Thermal Resistance across an Interface
Source: Polymers (Basel). 2021 Oct 28;13(21):3732. doi: 10.3390/polym13213732 (PMC8588352; doi:10.3390/polym13213732)
Supplement: Supplementary file 1 [file polymers-13-03732-s001.zip › polymers-1436093-supplementary.pdf]

Supplementary Materials

Molecular Structure Effect of a Self-Assembled Monolayer on Thermal Resistance across an Interface

Lijian Song [1], Youchen Zhang [1,*], Weimin Yang [1,2], Jing Tan [1,2] and Lisheng Cheng [1,2,*]

[1] College of Mechanical and Electrical Engineering, Beijing University of Chemical Technology, Beijing 100029, China; song_li_jian@163.com (L.S.); yangwm@mail.buct.edu.cn (W.Y.); tanj@mail.buct.edu.cn (J.T.)
[2] State Key Laboratory of Organic-Inorganic Composites, Beijing University of Chemical Technology, Beijing 100029, China
[*] Correspondence: zhangyc@mail.buct.edu.cn (Y.Z.); chengls@mail.buct.edu.cn (L.C.)

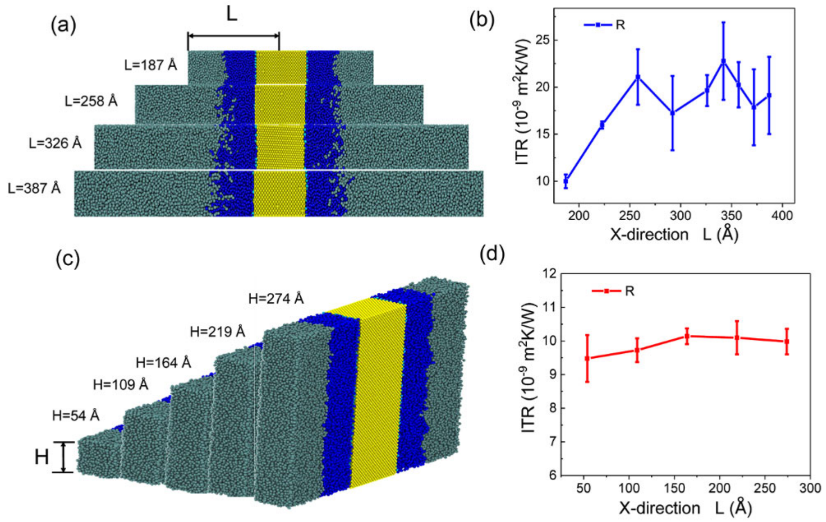

Figure S1. (a) Schematic representation under different lengths of the system. L is the half-length of the system. gold (yellow), PEG medium (cyan), PEG brush (blue). (b) Length effect on thermal resistance between PEG medium and PEG brush. After L reaches 326 Å, the ITR between PEG medium and PEG brush is stable. (c) Schematic representation of the system with different widths along the z direction. (d) Width effect on the thermal resistance between PEG medium and PEG brush. ITR showed few size effect along the width direction.

$$E = E_{\mathrm{bonded}} + E_{nonbonded}$$
$$= E_{angle} + E_{bond} + E_{dihed} + E_{vdw} + E_{\mathrm{coulomb}}$$
$$E_{\mathrm{vdw}} = 4\varepsilon[(\varepsilon / r)^{12} - (\varepsilon / r)^{6}]$$
$$E_{bond} = \sum_{bonds} K_{\mathrm{b}}(r - r_{eq})^2$$
$$E_{angle} = \sum_{angles} K_a(\theta - \theta_{eq})^2$$
$$E_{dihed} = \sum_{i=1,m} K_i[1 + \cos(n_i\phi - d_i)]$$

The total energy potential function comprises angle bending, bond stretching, and dihedral torsion terms for bonding interactions and LJ and coulomb terms for nonbonding interactions. In this study, coulomb interactions were not considered because the heat carriers were mainly phonons rather than electrons in polymer systems.

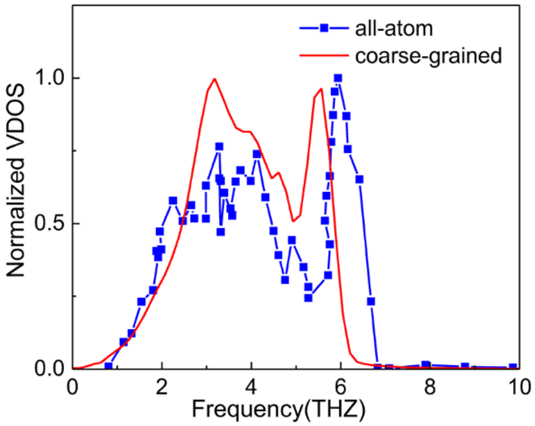

Figure S2. Normalized vibrational power spectral of Au substrate of all-atom model and our coarse-grained model.

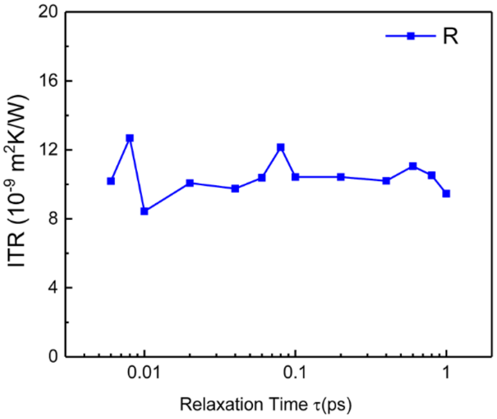

Figure S3. Effect of different relaxation times in Langevin thermal bath on ITR between PEG medium and PEG brush.

Table S1. Nonbonding interactions parameters of the system in the CG model.

Pair type	ε , kcal/mol	σ , Å
Au–Au	21.562	3.4
Au–SP_2	2.5	4.3
Au–COC	2.5	4.3
SP_2– SP_2	0.807	4.3
SP_2–SNO	0.717	4.3
COC–COC	0.6283	4.3

Table S2. Bonding parameters of the system in the CG model.

Bond type	K_b (kcal mol^{-1}Å^{-2})	r_{eq} (Å)
Au–SP_2	20.3155	3.8
SP_2–SNO	20.3155	3.3
COC–COC	20.3155	3.3
SNO-SNO	Variable	3.3

NOTE: Variable K_b of the brush: 2, 5, 10, 20, 30, 40, 60, 80, 120, 160, 240, 320, 480, and 640 kcal mol^{-1}Å^{-2}.

Table S3. Angle parameters of the system in the CG model.

Angle *type*	K_a (kcal mol^{-1})	θ_{eq} (°)
Au–SP_2–SNO	10.0000	180
SP_2–SNO-SNO	10.1577	130
COC-COC-COC	10.1577	130
SNO-SNO-SNO	10.1577	Variable

NOTE: Variable θ_{eq} of the brush: 100°, 110°, 120°, 130°, 140°, 150°.

Table S4. Dihedral parameters of the system in the CG model.

Dihedral type	K_1 (kcal/mol)	K_2 (kcal/mol)	K_3 (kcal/mol)	n_1	n_2	n_3	d_1 (°)	d_2 (°)	d_3 (°)
SP_2–SNO–SNO–SNO	0.468	0.043	0.0287	1	2	3	180	0	0
COC–COC–COC–COC	0.468	0.043	0.0287	1	2	3	180	0	0
